# Association between frailty index and cognitive dysfunction in older adults: insights from the 2011–2014 NHANES data

**DOI:** 10.3389/fnagi.2024.1458542

**Published:** 2024-09-05

**Authors:** Jianlong Zhou, Yadi Li, Lv Zhu, Rensong Yue

**Affiliations:** ^1^Hospital of Chengdu University of Traditional Chinese Medicine, Chengdu, China; ^2^Clinical Medical School, Chengdu University of Traditional Chinese Medicine, Chengdu, China; ^3^West China Center of Excellence for Pancreatitis, Institute of Integrated Traditional Chinese and Western Medicine, West China Hospital, Sichuan University, Chengdu, China

**Keywords:** cognitive dysfunction, frailty index, NHANES, older adults, restricted cubic spline regression model

## Abstract

**Background:**

As the population ages, the occurrence of cognitive decline and dementia is continuously increasing. Frailty is a prevalent problem among older adults. Epidemiologic studies have shown a comorbidity between frailty and cognitive impairment. However, their relationship remains unclear. The frailty index is an important indicator for measuring frailty. This study aims to investigate the relationship between frailty index and cognitive dysfunction in older adults aged 60 years and older in the United States from the 2011–2014 National Health and Nutrition Examination Survey (NHANES).

**Methods:**

Community-dwelling older adults aged 60 years or older from 2011 to 2014 were extracted from the NHANES database. The frailty index was calculated using the formula: frailty index = total number of deficits present/total number of deficits measured. The Animal Fluency (AF), the Digit Symbol Substitution Test (DSST), the Consortium to Establish a Registry for Alzheimer’s disease Delayed Recall (CERAD-DR), and Word Learning (CERAD-WL) were used to evaluate cognitive dysfunction. Firstly, weighted logistic regression analysis was used to explore the relationship between frailty index and cognitive dysfunction. Secondly, the influence of covariates on the frailty index was evaluated by subgroup analysis and interaction. Finally, the non-linear relationship is discussed by using the restricted cubic spline regression model.

**Results:**

Our study included a total of 2,574 patients, weighted logistic regression analysis, after adjusting for all covariates, showed that the frailty index was associated with every test score. The interaction showed that covariates had no significant effect on this association in AF. The association between the frailty index and AF in the restricted cubic spline regression model is non-linear. As the frailty index increased, the risk of AF reduction increased, suggesting a higher risk of cognitive dysfunction.

**Conclusion:**

In general, a high frailty index appears to be associated with an increased risk of cognitive dysfunction in the elderly. Consequently, protecting against cognitive decline necessitates making geriatric frailty prevention and treatment top priorities.

## 1 Introduction

Neurological disorders are the primary reason for disability and the secondary cause of mortality on a global scale. The occurrence of cognitive decline and dementia is continuously increasing, and it is expected that by 2,040, more than 1.9 million individuals across the globe will be affected by this condition (Ahmadi-Abhari et al., 2017). This not only has an impact on the patients themselves but also places a significant burden on their families and society as a whole (Matthews et al., 2019). We used a set of four cognitive tests to evaluate cognitive impairment: the Digit Symbol Substitution Test (DSST), Animal Fluency (AF), Consortium to Establish a Registry for Alzheimer’s disease Delayed Recall (CERAD-DR), and Word Learning (CERAD-WL) tests (Gong et al., 2021; Zhang et al., 2022). These tests were chosen to gauge cognitive function accurately. At present, the early detection and successful management of cognitive dysfunction remain somewhat restricted (Piersol et al., 2018). Therefore, researchers consider the prevention of cognitive dysfunction as a more feasible strategy (Liss et al., 2021; Zetterberg and Stomrud, 2021).

Frailty as a measure of deficit accumulation can capture health problems over the adult life course. Epidemiologic studies have shown a comorbidity between frailty and cognitive impairment (Vargas-Torres-Young et al., 2022). Current models for commonly measuring and understanding frailty are the Frailty Index (Rockwood and Mitnitski, 2007) and the phenotype of frailty (Fried et al., 2001). Frailty is a prevalent issue among older individuals, leading to increased risks of falls, disability, mobility issues, hospitalization, and diminished quality of life compared to their healthier counterparts (Chen et al., 2018). Consequently, it is imperative to acknowledge the vulnerability of this population and strive to prevent, delay, reverse, or minimize the adverse effects associated with frailty.

To the best of our knowledge, there are no studies on frailty index and cognitive dysfunction, and the National Health and Nutrition Examination Survey (NHANES), a well-designed, large-sample clinical registry database with complete follow-up, is well-positioned to discuss the association between frailty index and cognitive dysfunction, as well as the prognosis of cognitively dysfunctional patients. To supplement clinical studies, we utilized the NHANES database from 2010 to 2014 to investigate the correlation between frailty indexicality and cognitive dysfunction.

## 2 Methods

### 2.1 Study population

The NHANES database, including the National Health and Nutrition Examination Survey, is a comprehensive research database designed to assess and monitor the personal health and nutrition status of adults and children living in the United States. This program dates back to the early 1960s and initially focused on conducting surveys pertaining to various populations or specific health-related topics. Each year, NHANES selects a representative sample of approximately 5,000 individuals, selected from counties across the country. These participants are chosen to ensure a diverse representation of the population, in terms of demographics and geographic location. To this end, NHANES visits 15 different counties annually in order to collect data from a wide range of individuals. The survey component of NHANES involves conducting interviews that cover a variety of crucial aspects, including demographics, socioeconomic status, dietary habits, and relevant health-related inquiries. These interviews serve to gather valuable information about the individuals’ backgrounds and lifestyle choices, providing crucial data for analysis and further examination. The physical examination portion includes physiologic measurements and laboratory tests. The results of the survey are used to determine the prevalence of major diseases and risk factors for disease, and are the basis for national standards for height, weight, and blood pressure (Surveys will be conducted every 2 years, with 2 years referred to as a cycle year). Detailed NHANES study design and data are available at https://www.cdc.gov/nchs/nhanes/.

**Figure 1:**
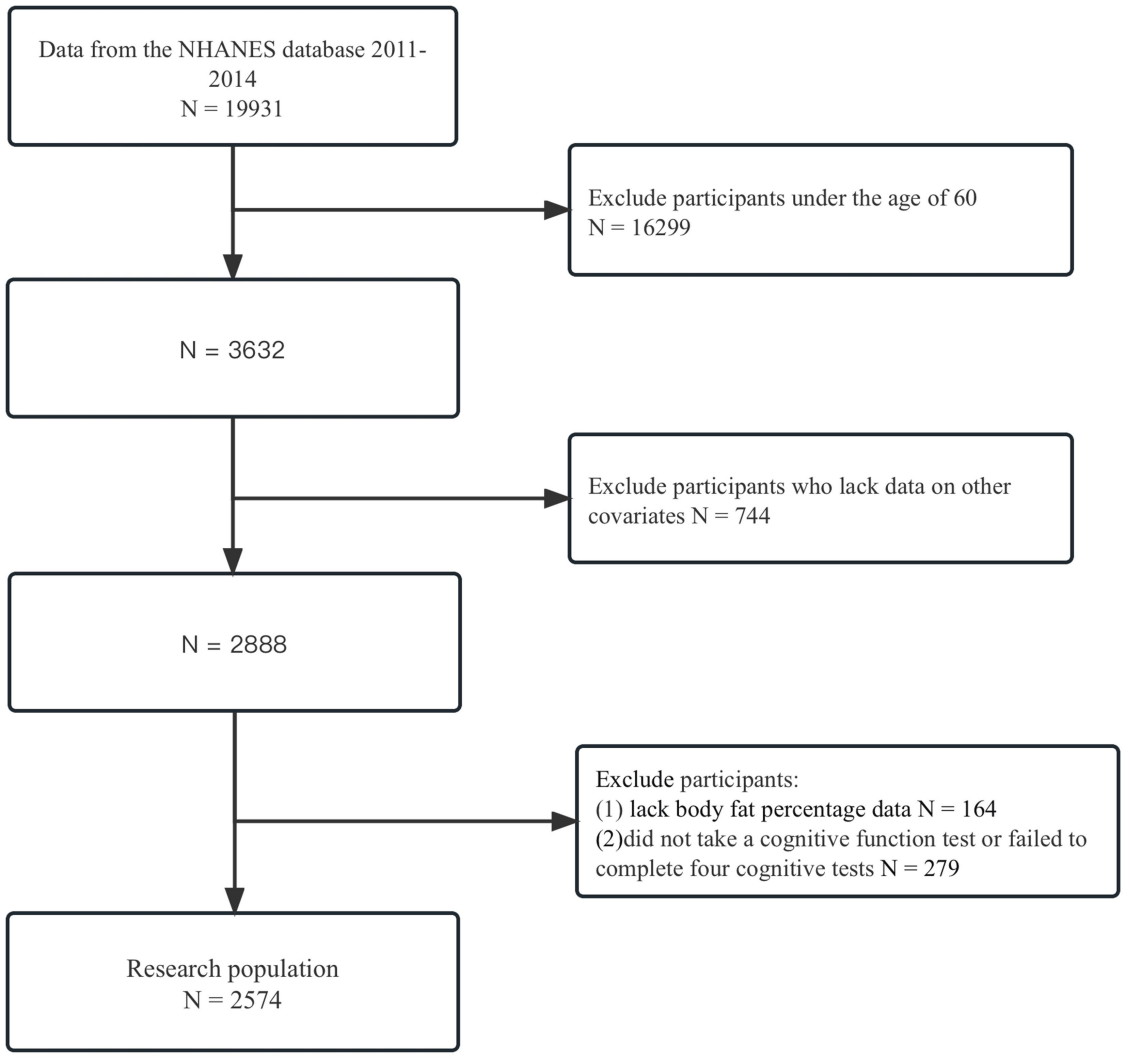
Flowchart of patient exclusion. The exclusion criteria were used to identify participants over 60 years of age who had completed a cognitive function score between 2000 and 2014, using data from the NHANES database.

In this study, we obtained data from a total of 19,931 individuals who participated in the NHANES survey between 2011 and 2014. To ensure the accuracy and validity of our analysis, we excluded respondents who were under the age of 60 (*N* = 16,299), resulting in a remaining sample of 3,632 individuals. Moreover, we also excluded respondents who had missing data for important variables such as gender, race, marital status, education, physical activity, the ratio of family income to poverty, smoked at least 100 cigarettes in life, at least 12 alcohol drinks/1 yr, hypertension, diabetes, and daily low-dose aspirin use. This exclusion resulted in a total of 744 respondents being excluded, leaving us with a final sample size of 2,888 individuals for our analysis. Additionally, participants who lacked information on the frailty index (*N* = 164; residual 2,724) were excluded, as well as those who did not undergo cognitive function tests or failed to complete the four cognitive tests (*N* = 279; residual 2,574). Finally, our study included a total of only 2,574 participants, as shown in Figure 1. This image displays the selection process.

### 2.2 The definition of cognitive dysfunction

Individuals who are 60 years of age and older meet the eligibility criteria for cognitive function assessments administered through various tests in NHANES. The tests used to assess Alzheimer’s disease include the establishment of the Alzheimer’s Registry Consortium (CERAD) test, as well as AF and DSST assessments. The CERAD test focuses specifically on vocabulary learning and memory, consisting of three consecutive learning phases and a delayed recall test phase. During each learning session, participants were asked to name a set of 10 unrelated words. After 8–10 min, the delayed recall test phase began, asking participants to recall as many previously learned words as possible. This comprehensive approach allows for the comprehensive evaluation of cognitive function in older individuals. This module is designed to test learners’ immediate and delayed learning of new words. Animal Fluency Test: Subjects are asked to list the names of animals in 1 min, with one point for each animal named. This module is designed to test the subject’s executive ability. Digital Symbol Substitution Test (DSST): a test with the numbers 1 through 9 and matching shapes at the top of the test. Subjects are asked to sequentially match the 133 numbers in the lower part of the test to the graphic in order within 2 min. The test is designed to examine subjects’ processing speed, sustained attention, and working memory. Higher scores on all tests indicate better cognitive abilities. The evaluation is conducted by skilled interviewers during the private, face-to-face interview at the Mobile Testing Center. The National Health and Nutrition Examination Survey (NHANES), available at https://www.cdc.gov/nchs/nhanes/index.htm, provides a comprehensive overview of the cognitive functioning test, including aspects such as quality assurance, quality control, data processing, and editing. In order to distinguish potential cognitive dysfunction from healthy cognitive function, this study adopts cutoff values supported by previously published literature (Bailey et al., 2020): AF less than 14, DSST less than 34, CERAD-WL less than 17, and CERAD-DR less than 5.

### 2.3 The definition of frailty index

The frailty index is a composite of physical, functional, psychological, and social health variables. The frailty index scale consists of 70 health deficiencies in these four areas, each of which is a deficiency that is scored as “1” when it is present and “0” when it is absent. The formula for calculating the frailty index is frailty index = total number of defects present/total number of defects measured. Frailty index ≥ 0.25 suggests that the elderly person is frail; Frailty index: 0.12–0.25 is pre-frailty; Frailty index < 0.12 is no frailty (Hoover et al., 2013).

### 2.4 Covariates

The covariates analyzed in our study that may potentially influence the occurrence of cognitive dysfunction encompass various factors such as age, gender (male or female), race (categorized as Mexican American, non-Hispanic white, non-Hispanic black, other Hispanic, or other race), educational background (< 11th grade, high school graduate, some college or AA degree, and college graduate or above), Ratio of family income to poverty (<1, 1–2, 2–5; Gong et al., 2021). Other aspects taken into consideration were physical activity (whether the individual engages in it or not), smoked at least 100 cigarettes in life (Yes or No), at least 12 alcohol drinks/1 yr (Yes or No), hypertension (Yes or No), diabetes (Pre-diabetes, Diabetes, No), Daily low-dose aspirin use (Yes or No).

### 2.5 Statistical analysis

Since the NHANES data were surveyed using complex multistage sampling, we selected “WTMEC2YR” for 2011–2014 and calculated these weights using the following formula:


$$\mathrm{wt}=\frac{1}{2}*\mathrm{WTMEC}2YR$$

Regarding the data expressed as unweighted counts (weighted %) for categorical variables, the mean (standard deviation) for continuous variables. In order to facilitate the test and comparison between different groups, we used the chi-square test for categorical variables and T-test for continuous variables. This approach allowed us to carefully analyze and contrast the various groups with accuracy and precision. This allowed us to gather valuable insights and draw meaningful conclusions from our research. The frailty index was categorized into tertiles from the lowest (T1) to the highest (T3). In order to explore the association between cognitive dysfunction and frailty index, weighted univariate logistic regression analyses were first performed. Second, the crude model did not account for covariates. However, in Model 1, the analysis was adjusted for variables such as gender, age, and race. In model 2, adjustments were made for age, gender, race, ratio of family income to poverty, education, hypertension, diabetes, low-dose aspirin in use, alcohol use, smoking, and physical activity. The association between frailty index and cognitive dysfunction was analyzed using weighted multivariate logistic regression models. To better characterize the non-linear dose–response relationship between frailty index and cognitive dysfunction, we used a restricted cubic spline regression model. We conducted a thorough examination and segmentation of interactions based on various factors such as age, gender, race, socioeconomic status, education level, hypertension, diabetes, history of low-dose aspirin intake, alcohol consumption, smoking habits, and physical activity. It is important to note that all statistical evaluations were conducted using a two-sided approach and assessed at a significance level of 0.05. All analyses were performed using R (version 4.2.3. http://www.r-project.org) was performed.

## 3 Results

### 3.1 Baseline characteristics of the study population

Table 1 demonstrates the pertinent characteristics of the individuals participating in the study. In total, the study encompassed 2,574 older adults with an age of 60 years or above. The average frailty index, represented as the mean (SD), was found to be 0.17 (0.00). Furthermore, the prevalence of cognitive impairment was evaluated through various assessments. The weighted prevalence for the Digit Symbol Substitution Test (DSST) was calculated to be 22.12%, while it was 21.42% for the Consortium to Establish a Registry for Alzheimer’s Disease Word List Learning (CERAD-WL). Moreover, the CERAD Delayed Recall (CERAD-DR) assessment revealed a prevalence of 21.34%, and the Animal Fluency (AF) test resulted in a prevalence of 20.77%. The clinical characteristics of the participants, based on the frailty index as a column-stratified variable, are shown in. From it, we can find statistically significant differences (*p* < 0.05) in gender, race, education, the ratio of family income to poverty, at least 12 alcohol drinks/1 yr, smoked at least 100 cigarettes in life, hypertension, and diabetes.

**Table 1:** Study population characteristics by tertiles of frailty index.

Variable	Overall	Tertile 1	Tertile 2	Tertile 3	*p* value
Frailty Index	0.17 (0.00)	0.08 (0.01)	0.16 (0.03)	0.31 (0.02)	<0.001
Age (year)	69.09 (0.20)	67.34 (0.27)	69.69 (0.25)	70.99 (0.46)	<0.001
DSST < 40 (%)	22.12	10.15	20.94	41.85	<0.001
CERAD-WL < 17 (%)	21.42	14.03	23.85	29.6	<0.001
CERAD-DR < 5 (%)	21.37	14.78	21.08	31.78	<0.001
AF < 14 (%)	20.77	13.91	19.43	32.94	<0.001
Gender (%)					0.01
Female	53.91	51.34	52.07	60.19	
Male	46.09	48.66	47.93	39.81	
Race/Hispanic origin (%)					0.005
Mexican American	3.14	2.73	2.77	4.23	
Non-Hispanic Black	7.99	5.78	9.08	9.97	
Non-Hispanic White	80.53	83.01	79.21	78.45	
Other Hispanic	3.46	3.2	3.57	3.69	
Other Race	4.88	5.28	5.36	3.66	
Marital status (%)					<0.001
Married	62.01	68.29	63.35	50.74	
Never married	4.33	4.15	4.1	4.89	
Others	33.66	27.56	32.55	44.37	
Education (%)					<0.001
< 11^th^ grade	15.62	9.79	14.35	26.14	
High school graduate	20.44	18.43	20.57	23.34	
Some college or AA degree	31.73	31.44	34.05	29.2	
College graduate or above	32.21	40.33	31.03	21.32	
Ratio of family income to poverty (%)					<0.001
< 1	8.97	4.8	9.57	14.55	
1–2	23.57	17.4	21.59	35.49	
2–5	67.47	77.79	68.84	49.96	
At least 12 alcohol drinks/1 yr (%)					0.011
No	26.85	23	29.42	29.43	
Yes	73.15	77	70.58	70.57	
Smoked at least 100 cigarettes in life (%)					0.013
No	49.09	54.48	46.77	43.85	
Yes	50.91	45.52	53.23	56.15	
Hypertension (%)					<0.001
No	40.75	62.76	31.15	19.47	
Yes	59.25	37.24	68.85	80.53	
Diabetes (%)					<0.001
Pre-diabetes	4.24	3.04	4.57	5.65	
Diabetes	19.58	3.78	23.59	38.54	
No	76.18	93.18	71.84	55.82	
Daily low-dose aspirin use (%)					<0.001
No	46.25	55.82	42.26	36.77	
Yes	53.75	44.18	57.74	63.23	
Physical activity (%)					0.232
No	7.79	9.33	6.72	6.81	
Yes	92.21	90.67	93.28	93.19	

Mean (SD) for continuous variables: *p* values were calculated using a weighted *t* test. % is a categorical variable: the *p*-value is calculated using a weighted chi-square test.

### 3.2 Univariate logistic regression analysis of CD

On the basis of univariate logistic regression (Table 2), it can be concluded that there was an increased risk for cognitive function test scores among those who were older (> 60 years), non-Hispanic blacks, other Hispanics, other marital status, female, drinking alcohol, diabetes (yes), and hypertension (yes; OR > 1, *p* < 0.05). However, participants of Mexican American, other race, ratio of family income to poverty (>1), and higher education demonstrated a decreased risk for cognitive function test scores (OR < 1, *p* < 0.05).

**Table 2:** Weighted univariate logistic analysis of cognitive function.

Variable	DSST < 40		CERAD-WL < 17		CERAD-DR < 5		AF < 14	
	OR 95% CI	*p*-value	OR 95% CI	*p*-value	OR 95% CI	*p*-value	OR 95% CI	*p*-value
Frailty Index	1.08 (1.07,1.10)	<0.001	1.10 (1.08,1.13)	<0.001	1.04 (1.03,1.05)	<0.001	1.05 (1.04,1.07)	<0.001
Age (year)	1.11 (1.09,1.13)	<0.001	1.04 (1.03,1.05)	<0.001	1.12 (1.09,1.14)	<0.001	1.08 (1.06,1.09)	<0.001
Gender								
Female								
Male	1.17 (0.90,1.52)	0.23	1.47 (1.17,1.83)	0.002	1.42 (1.11,1.83)	0.011	0.90 (0.69,1.17)	0.402
Race/Hispanic origin								
Mexican American								
Non-Hispanic Black	1.00 (0.70,1.44)	0.993	0.76 (0.49,1.20)	0.241	0.87 (0.57,1.32)	0.49	1.87 (1.32,2.65)	<0.001
Non-Hispanic White	0.21 (0.16,0.28)	<0.001	0.52 (0.33,0.80)	0.005	0.66 (0.44,0.98)	0.043	0.53 (0.37,0.75)	<0.001
Other Hispanic	1.35 (0.82,2.22)	0.23	1.24 (0.85,1.81)	0.251	1.01 (0.68,1.50)	0.952	1.49 (1.00,2.20)	0.052
Other Race	0.22 (0.13,0.38)	<0.001	0.67 (0.39,1.15)	0.143	0.51 (0.29,0.89)	0.022	1.29 (0.74,2.26)	0.366
Marital status								
Married								
Never married	1.46 (0.84,2.53)	0.173	1.28 (0.77,2.12)	0.334	0.81 (0.45,1.46)	0.481	1.42 (0.82,2.46)	0.201
Others	1.93 (1.56,2.40)	<0.001	1.56 (1.24,1.97)	<0.001	1.27 (0.98,1.65)	0.075	1.46 (1.10,1.93)	0.018
Education								
< 11^th^ grade								
High school graduate	0.37 (0.25,0.54)	<0.001	0.65 (0.46,0.93)	0.019	0.91(0.65,1.26)	0.558	0.80 (0.57,1.11)	0.183
Some college or AA degree	0.18(0.13,0.26)	<0.001	0.38 (0.25,0.57)	<0.001	0.45 (0.32,0.62)	<0.001	0.38 (0.31,0.46)	<0.001
College graduate or above	0.10 (0.07,0.15)	<0.001	0.27 (0.17,0.42)	<0.001	0.44 (0.29,0.69)	<0.001	0.25 (0.18,0.34)	<0.001
Ratio of family income to poverty								
< 1								
1–2	0.55 (0.43,0.69)	<0.001	0.66 (0.51,0.86)	<0.001	1.04 (0.79,1.37)	0.794	0.61 (0.45,0.83)	<0.001
2–5	0.15 (0.10,0.20)	<0.001	0.28 (0.22,0.37)	<0.001	0.46 (0.33,0.65)	<0.001	0.31 (0.20,0.48)	<0.001
At least 12 alcohol drinks/1 yr								
No								
Yes	0.47 (0.40,0.55)	<0.001	0.61 (0.50,0.75)	<0.001	0.71 (0.57,0.89)	0.004	0.54 (0.46,0.65)	<0.001
Smoked at least 100 cigarettes in life								
No								
Yes	1.24 (0.99,1.56)	0.063	0.89 (0.73,1.08)	0.233	0.81 (0.64,1.02)	0.071	0.98 (0.81,1.19)	0.861
Hypertension								
No								
Yes	2.07 (1.58,2.70)	<0.001	1.44 (1.09,1.91)	0.017	1.40 (1.10,1.80)	<0.001	1.59 (1.25,2.02)	<0.001
Diabetes								
Pre-diabetes								
Diabetes	2.27 (1.23,4.17)	0.011	1.73 (0.96,3.10)	0.07	0.93 (0.53,1.65)	0.7	1.97 (0.94,4.11)	0.076
No	1.03 (0.60,1.79)	0.918	1.24 (0.72,2.12)	0.422	0.78 (0.46,1.33)	0.352	1.24 (0.68,2.25)	0.476
Daily low-dose aspirin use								
No								
Yes	1.17 (0.93,1.47)	0.185	1.12 (0.95,1.32)	0.174	1.20 (0.96,1.51)	0.114	1.23(0.96,1.59)	0.105
Physical activity								
No								
Yes	0.83 (0.59,1.18)	0.293	0.91 (0.59,1.40)	0.65	0.98 (0.59,1.60)	0.923	0.74 (0.51,1.06)	0.107

AF, the animal fluency; CERAD-DR, the consortium to establish a registry for Alzheimer’s disease delayed recall; CERAD-WL, the consortium to establish a registry for Alzheimer’s disease word learning; DSST, digit symbol substitution test. 95% CI, 95% confidence interval; OR: odds ratio; *p* < 0.05 was considered statistically significant.

### 3.3 Association between frailty index and CD

Overall, in DSST, CERAD-WL, CERAD-DR, and AF scores, the results of the three models were similar, with Odds ratio (OR) attenuating whenever more covariates were included in the model (OR < 1, *p* < 0.05). This demonstrated a significant correlation between the frailty index and cognitive function scores. We further converted the frailty index from a continuous variable to a categorical variable (tertiles) for sensitivity analysis (Table 3). It was found that in the same model in higher frailty index subgroups, ORs increased (OR > 1, *p* < 0.05). However, the positive correlation between frailty index and CD became negligible in Models 1 and 2 (Table 3).

**Table 3:** Weighted multivariate logistic analysis frailty index and cognitive function.

Cognitive test	Frailty Index		T1		T2		T3	
DSST < 40 (%)	OR (95% CI)	*p*-value	OR (95% CI)	*p*-value	OR (95% CI)	*p*-value	OR (95% CI)	*p*-value
Crude model	1.08 (1.07,1.10)	<0.001			2.34 (1.78,3.08)	<0.001	6.37 (4.55,8.92)	<0.001
Model 1	1.08 (1.06,1.09)	<0.001	Ref		1.87 (1.44,2.44)	<0.001	5.36 (3.89,7.39)	<0.001
Model 2	1.07 (1.05,1.08)	<0.001			1.66 (1.21,2.26)	0.005	3.87 (2.51,5.95)	<0.001
CERAD-WL < 17 (%)								
Crude model	1.04 (1.03,1.05)	<0.001			1.92 (1.51,2.45)	<0.001	2.58 (2.01,3.30)	<0.001
Model 1	1.03 (1.02,1.04)	<0.001	Ref		1.56 (1.21,2.02)	0.001	1.94 (1.53,2.47)	<0.001
Model 2	1.02 (1.01,1.04)	<0.001			1.53 (1.08,2.16)	0.028	1.62 (1.18,2.23)	0.019
CERAD-DR < 5 (%)								
Crude model	1.04 (1.03,1.05)	<0.001			1.54 (1.19,1.99)	0.002	2.69 (1.88,3.85)	<0.001
Model 1	1.03 (1.02,1.04)	<0.001	Ref		1.12 (0.93,1.54)	0.14	1.96 (1.37,2.81)	<0.001
Model 2	1.03 (1.02,1.04)	<0.001			1.24 (0.93,1.67)	0.13	1.91 (1.24,2.95)	0.016
AF < 14 (%)								
Crude model	1.05 (1.04,1.07)	<0.001			1.49 (1.06,2.11)	0.022	3.04 (2.09,4.42)	<0.001
Model 1	1.04 (1.03,1.06)	<0.001	Ref		1.20 (0.82,1.75)	0.323	2.34 (1.56,3.50)	<0.001
Model 2	1.04 (1.03,1.06)	0.004			1.15 (0.75,1.75)	0.02	2.04 (1.26,3.32)	0.015

AF, the animal fluency; CERAD-DR, the consortium to establish a registry for Alzheimer’s disease delayed recall; CERAD-WL, the consortium to establish a registry for Alzheimer’s disease word learning; DSST, digit symbol substitution test. Crude model, no covariates were adjusted. Model 1, age, gender, and race were adjusted. Model 2, age, sex, race, ratio of family income to poverty, education, hypertension, diabetes, daily low-dose aspirin use, smoked at least 100 cigarettes in life, at least 12 alcohol drinks/1 yr, and physical activity were adjusted. 95% CI, 95% confidence interval; OR: odds ratio; *p* < 0.05 was considered statistically significant.

### 3.4 Subgroup analysis and interaction effects

**Figure 2:**
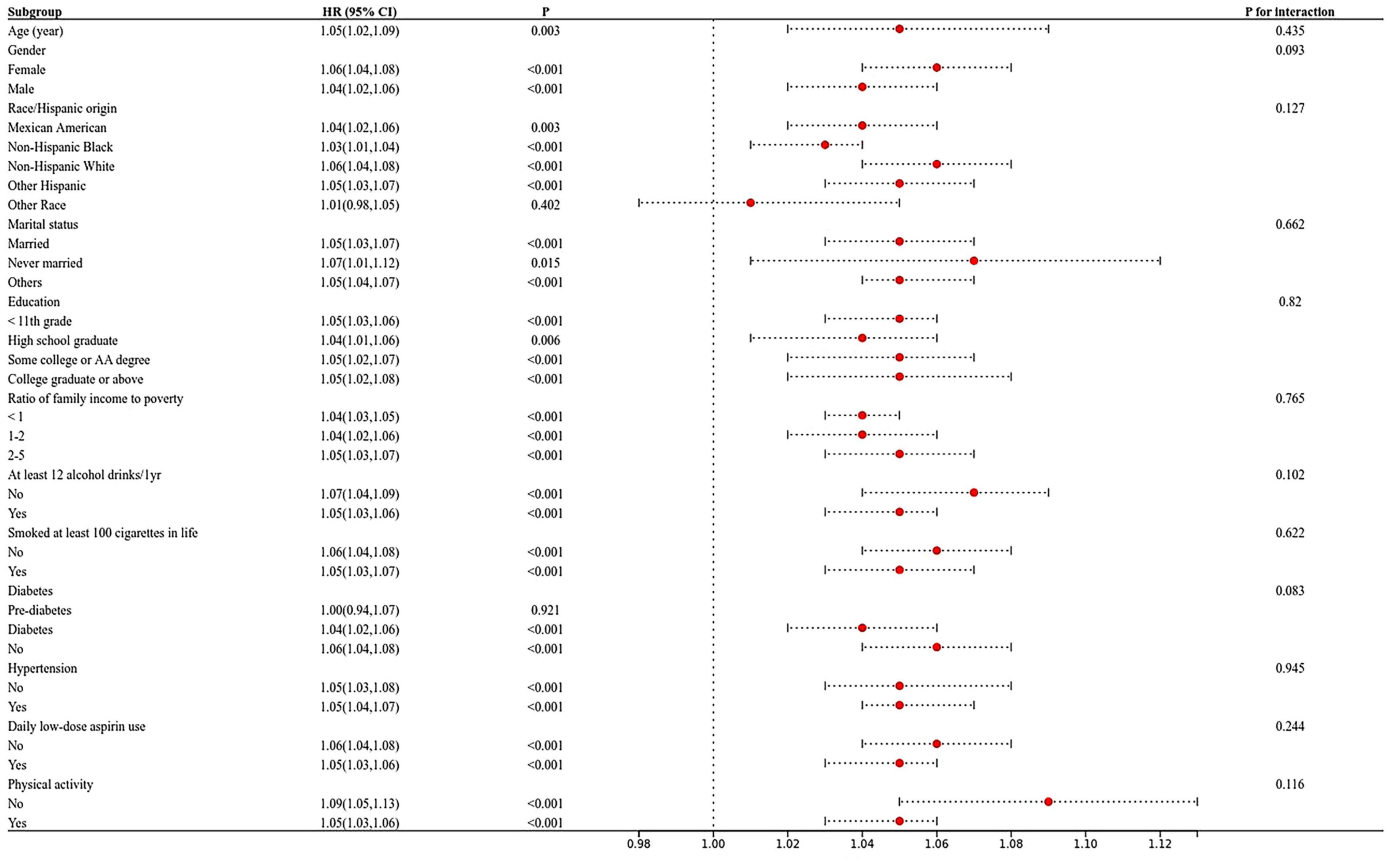
The relationship between frailty index and cognitive function in the elderly was analysed by forest map stratification. *p* for interaction represents the influence of age, gender, race, marital status, the ratio of family income to poverty, education, smoking, alcohol drinks, hypertension, diabetes, low-dose aspirin use, and physical activity on the association between body fat percentage and cognitive function, *p* < 0.05 indicates an impact.

Subgroup analysis in which all variables were stratified was shown in Figure 2. Overall, this correlation was statistically significant (*p* > 0.05) for participants with age, gender, race, marital, education level, the ratio of family income to poverty, physical activity, smoked at least 100 cigarettes in life, at least 12 alcohol drinks/1 yr, hypertension, diabetes, and daily low-dose aspirin use.

Subsequently, we performed interaction findings for each variable, and the results showed that the relationship between frailty index and AF score tests was not statistically different across classes, suggesting that age, gender, marital, education, physical activity, smoked at least 100 cigarettes in life, at least 12 alcohol drinks/1 yr, hypertension, diabetes, and daily low-dose aspirin use did not significantly affect this positive association. For DSST, race, marital status, education level, income-to-poverty ratio, hypertension, and diabetes in the model, all interacted on the frailty index (Figure 2).

### 3.5 The non-linear relationship between frailty index and CD

**Figure 3:**
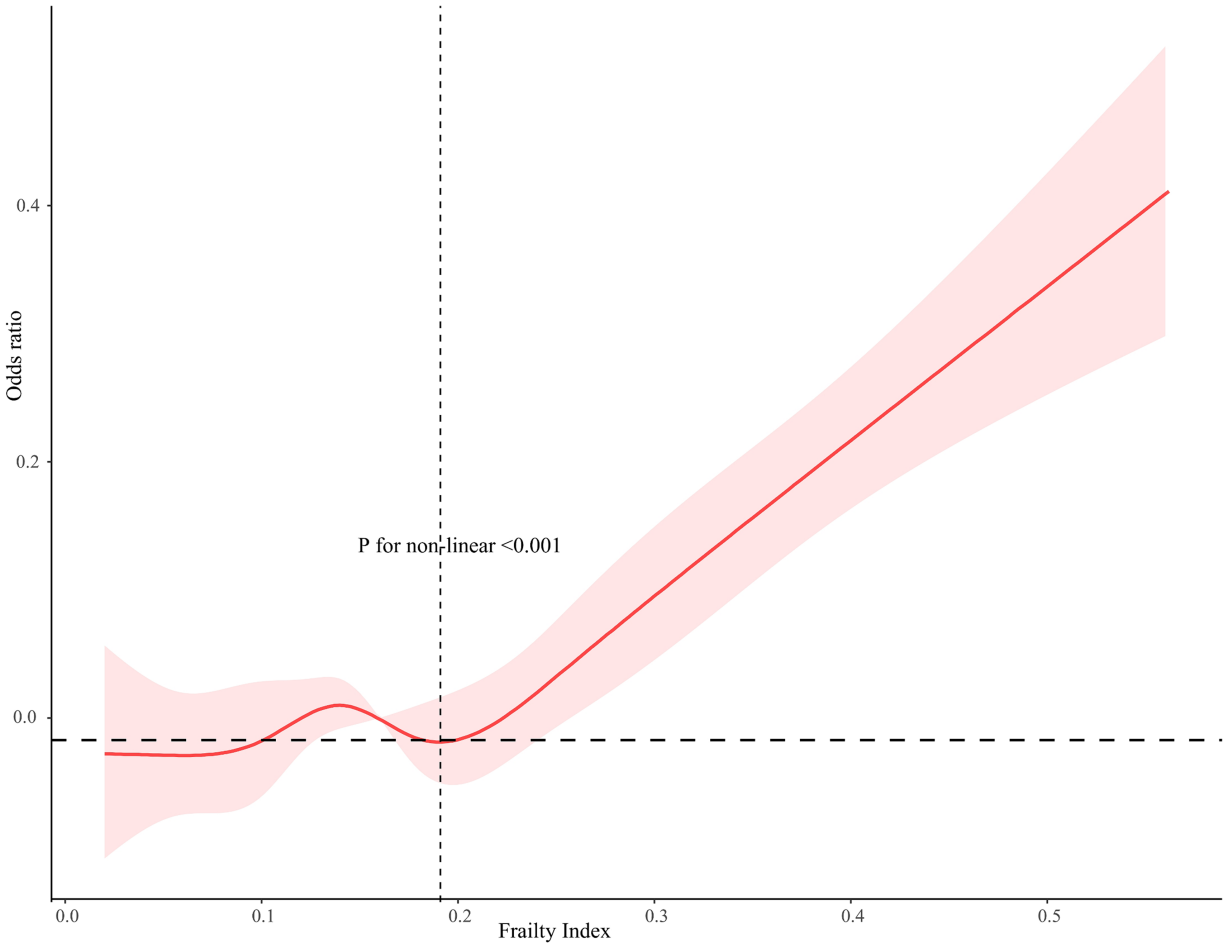
Restricted cubic spline relationship between frailty index and the risk of cognitive function. Models adjusted for age, gender, race, marital status, ratio of family income to poverty, education, smoking, alcohol drinks, hypertension, diabetes, low-dose aspirin use, and physical activity. The dashed area indicates 95% CI.

As Figure 3 shown, a non-linear dose–response relationship was found between the frailty index and AF, using restricted cubic splines (AF, *p* for non-linear <0.001). For AF, the risk of low cognitive performance started to increase when the frailty index was higher than 0.190 (Figure 3). The frailty index was statistically significantly associated with an elevated risk of low cognitive performance in AF when the frailty index.

## 4 Discussion

This research aims to examine the correlation between the frailty index and cognitive function in individuals aged 60 years or older. The study involves a retrospective analysis of cognitive dysfunction data collected from the NHANES database between 2011 and 2014. Older adults in the highest tertile were at higher risk for low cognitive function. The study found that the frailty index was significantly associated with CERAD-DR/AF score tests, even after adjusting for multiple sociodemographic, laboratory data, questionnaire, and examination data covariates, and even after controlling for full potential confounders.

To the best of our knowledge, our study reported finding that the frailty index was associated with lower cognitive scores. Our results are consistent with previous studies. Shu et al. found that older people who are frail are more likely to have cognitive decline (OR: 5.76, 95% confidence interval: 1.20–27.6) and memory decline (OR: 5.53, 95% confidence interval: 1.64–18.7) than older people who are not frail (Nishiguchi et al., 2015). Kota et al. found that social vulnerability was associated with every cognitive deficit (OR = 1.61, 95%CI: 1.13–2.30) and physical functioning deficits (OR = 1.99, 95%CI: 1.57–2.52) independently (Tsutsumimoto et al., 2017). A meta-analysis found statistically significant differences in both comparisons when it came to cognitive status scores for participants in the non-vulnerable (*N* = 12,729, 47.4%) versus pre-fragile (*N* = 11,559, 43.2%) and non-vulnerable versus fragile (*N* = 2,452, 9.4%) subgroups (M ± SD = 0.60, 95% CI: 0.50–0.62, *p* < 0.001 and M ± SD = 3.43, 95% CI: 2.26–4.60, *p* < 0.001; Furtado et al., 2018). In another meta-analysis, results from cross-sectional studies of cognitive performance in frail and non-frail or able-bodied older adults (60+ years of age) indicated that in both the Global Test of Cognitive Functioning (OR = 0.734: 95% CI: 0.601–0.867) and the Individual Cognitive Domains (OR = 0.439: 95% CI: 0.342–0.535) tests, the frailty status had a functioning had an overall significant negative effect (Robinson et al., 2022).

The connection between levels of frailty and various diseases has been examined in past epidemiological studies. Notably, Ma et al. discovered a notable association between the frailty index and age. Additionally, it was found that the prevalence of frailty was considerably higher in individuals with hypertension compared to those without the condition (Ma et al., 2020). A study of 314,093 participants in the UK Biobank found an absolute prevalence difference of 1.67 (95% CI: 1.33–2.02) for pre-frailty and 5.00 (95%: 4.03–5.97) for frailty compared to a non-frail population with an overall cardiovascular disease prevalence rate of 6.54% (Chen et al., 2023). Frisoli et al. found that frailty was an independent predictor of disability [HR: 3.94 (1.59–9.75) *p* = 0.003] and it tripled the probability of death compared to the robust group. In conclusion, elderly outpatients with CVD are more frail than those without CVD (Frisoli et al., 2015). A study of older Spanish adults showed that frailty was associated with incident disability or mortality (HR 3.3; 95%CI: 1.7–6.6) adjusted for all study covariates (de la Rica-Escuín et al., 2014). In addition, one study found that the prevalence of frailty and premorbidity was higher in older adults with diabetes than in those without diabetes (Tang et al., 2013; Chhetri et al., 2017; Lyu et al., 2023). One study found that the prevalence of frailty and pre-frailty was very high in Chinese older adults with asthma, and assessment of frailty should become routine in the treatment of older adults with asthma (Huang et al., 2019; Zeng et al., 2023). However, there are no direct studies demonstrating whether the frailty index is associated with low cognitive function.

The mechanisms by which the frailty index increases the likelihood of cognitive decline are complex. First, the frailty index is calculated from a composite score and may involve a disease associated with multiple physiological systems. Previous studies have shown that the frailty index is associated with a variety of diseases such as hypertension, diabetes, heart disease, and respiratory system (Tang et al., 2013; Frisoli et al., 2015; Ma et al., 2020; Zeng et al., 2023), which may affect the quality of their nutritional intake, lethargy, loss of appetite, dysbiosis, and even malabsorption, and ultimately increase the risk of malnutrition and lead to cognitive dysfunction. Another pathway may be a psychosocial phenomenon, some studies have found that frail older adults have a higher probability of fracture and disability in daily life (Kojima, 2016, 2017), this can lead to a prolonged inability to move around autonomously, once older adults stay at home for long periods of time instead of engaging in socialization activities they are at a high risk of developing depression. A study of Japanese older adults followed for 4 years found that the prevalence of depressive symptoms was 7.2%. Among them physical weakness 9.6%, cognitive impairment 9.3%, and social weakness 12.0% (Tsutsumimoto et al., 2018). Wei J et al. found that cognition in older adults may be altered by late-life depression (Wei et al., 2019). Frailty may increase the risk of low cognition through psychosocial effects such as depression. The last possible link between frailty and low cognitive function may be the inflammatory pathway, It was found that white blood cell (WBC), neutrophil (NE), neutrophil-to-lymphocyte ratio (NLR), neutrophil-to-albumin ratio (NAR), systemic immune-inflammation index (SII), systemic immune response index (SIRI), and dementia inflammation index (DII) scores were significantly higher in the cognitively impaired group compared to the normal group (*p* < 0.05). Furthermore, there was a positive correlation between cognitively impaired patients and systemic inflammatory indices (*p* < 0.05; Li et al., 2023). Previous studies have found that the levels of pro-inflammatory cytokines (especially IL-6 and IL-8), TNF-α, and CRP are significantly elevated in the frail state (Lai et al., 2014; Hammami et al., 2020), and if these mediators live in the brain, they may stimulate microglia in the brain and lead to a malignant inflammatory cycle, which ultimately damages the important neurons and leads to the decline of cognitive function (Magalhães et al., 2018).

Interestingly, the World Health Organization introduced the concept of intrinsic capacity (IC), defined as “the combination of all the physical and mental capacities of an individual.” Recent research suggested that IC may be a crucial factor in assessing an individual’s aging process, and that it is closely related to the progression of Alzheimer’s disease across its five dimensions (locomotion, vitality, sensory, cognitive, and psychosocial; López-Ortiz et al., 2024). Additionally, cognitive frailty is also a notable concept. It not only refers to cognitive impairment in those with co-morbid physical fragility but is also associated with a higher risk of dementia (Kocagoncu et al., 2022). These findings provide new insights into better understanding the concept of cognitive frailty in dementia.

DSST, CERAD-WL, CERAD-DR, and AFT were chosen as the research method because these tests are widely used to assess cognitive function, have high reliability and validity, and can fully reflect the cognitive ability of the subjects (Low et al., 2019). DSST is a commonly used cognitive function test to assess information processing speed and visual-motor skills, which requires subjects to complete as many symbol substitution tasks as possible within a certain period of time, and through this test, we can learn about the subjects’ abilities in attention, visual perception, and motor coordination (Auer et al., 2016). The CERAD test consists of several sub-tests such as the immediate recall and delayed recall tests for assessing memory functioning, especially for recent and distant memory, which are sensitive to assessing changes in cognitive functioning (Karrasch et al., 2005; Bailey et al., 2020). The AFT, on the other hand, is considered to reflect executive functions, such as semantic memory (Monteiro-Junior et al., 2022). It assesses a subject’s verbal organization and expressiveness by asking them to list the names of as many animals as possible starting with a specific letter within a certain time period (Wendell et al., 2016; Bailey et al., 2020). These tests were chosen based on their ability to comprehensively assess multiple aspects of cognitive functioning, including attention, memory, language ability, and speed of information processing, thereby more accurately reflecting the overall cognitive state of the subject. In addition, these test methods have been widely validated and applied with high reliability and validity, and are therefore considered appropriate choices in cognitive function research.

Our study contains many strengths. First, after searching the PubMed database, our report explored the link between frailty and cognitive functioning, and our results could bring more attention to the link between frailty indices and cognitive frailty. Additionally, our study involved the evaluation of cognitive impairment using a battery of four widely utilized individual tests. To ensure the reliability and accuracy of our findings, we took into account various covariates. These covariates were meticulously analysed using weighted logistic regression models, considering the complex sampling design of the NHANES database. By adjusting for these covariates and considering the multi-stage sampling data, we aimed to enhance the precision and dependability of our study’s conclusions. Furthermore, we employed restricted cubic splines and smooth curve fitting techniques to meticulously explore the potential nonlinear associations between variables. Finally, we assessed whether the covariates had an effect on the findings through subgroup analyses and interactions. The main results found a positive association between frailty index and low cognitive functioning.

However, there may be limitations to the results of the study, and we need to be cautious in making recommendations to guide clinical practice. First, it is important to note that this particular study was conducted using a retrospective design, which means that the study relied on past data, limiting the ability to establish a clear causal relationship between frailty and cognitive dysfunction. It is plausible that a bidirectional association exists between the frailty index and cognitive dysfunction among older individuals. This means that lower cognitive function in older adults could be attributed to a variety of factors, such as the presence of other underlying health conditions or a decline in self-care ability. Consequently, these factors may contribute to higher scores on the frailty index, thus creating the appearance of an elevated frailty level. Second, the limitations of retrospective studies may lead to missing data or incomplete cognitive tests, which may introduce selection bias, and thus the final sample may not be fully representative of the broader older population. Third, the cross-sectional nature of the study, capturing data at a single point in time, limits the ability to track changes in frailty and cognitive function over time. Longitudinal studies would be needed to understand how these conditions evolve and interact in the long term. Fourth, despite controlling for many variables, the study may still be influenced by residual confounders. Some confounders based on self-reported data, such as smoking and alcohol consumption, did not have accurate cigarette and alcohol consumption. Thus, although we have adjusted for them in our analyses, it may lead to recall bias. Finally, while the cognitive tests used in the study are validated and widely accepted, they may not capture all aspects of cognitive functioning, especially in diverse populations with different educational and cultural backgrounds. This reliance on standardized tests could overlook important cognitive domains that might provide a more comprehensive picture of cognitive health. They do, however, serve a valuable purpose in exploring the connection between cognitive function and various medical conditions and risk factors recorded in the NHANES database. To establish more precise correlations, it is crucial to conduct further research on this matter.

## 5 Conclusion

In our study, the frailty index was found to be associated with poorer cognitive functioning in older adults. This may be because cognitive abilities like processing speed, attention span, and working memory deteriorate with age. In a clinical setting, it’s crucial for clinicians to check for frailty in older people and deal with it as part of their clinical review. Identifying and intervening with patients who exhibit signs of frailty can help prevent the onset of cognitive impairment in the aging population. Additionally, healthcare providers should consider implementing customized screening tools to evaluate cognitive function specifically in frail older patients. This approach enables early detection and intervention for cognitive impairment, promoting overall well-being in the elderly.

## Data Availability

The original contributions presented in the study are included in the article/supplementary material, further inquiries can be directed to the corresponding author.
